# Mass deworming for soil‐transmitted helminths and schistosomiasis among pregnant women: A systematic review and individual participant data meta‐analysis

**DOI:** 10.1002/cl2.1052

**Published:** 2019-09-24

**Authors:** R. A. Salam, S. Cousens, V. Welch, M. Gaffey, P. Middleton, M. Makrides, P. Arora, Z. A. Bhutta

**Affiliations:** ^1^ Healthy Mother, Babies and Children Theme South Australian Health and MedicalResearch Institute Adelaide Australia; ^2^ Paediatrics and Reproductive Health University of Adelaide Adelaide Australia; ^3^ Maternal Adolescent Reproductive & Child Health (MARCH) Centre London School of Hygiene and Tropical Medicine London UK; ^4^ School of Epidemiology, Public Health and Preventive Medicine University of Ottawa Ottawa Canada; ^5^ Centre for Global Child Health The Hospital for Sick Children Toronto Canada; ^6^ Robinson Research Institute University of Adelaide Adelaide Australia; ^7^ Dalla Lana School of Public Health University of Toronto Toronto Canada; ^8^ Centre of Excellence in Women and Child Health The Aga Khan University Karachi Pakistan

## Abstract

The objective of the review is to use individual participant data (IPD) meta‐analysis to explore the effect of mass deworming during pregnancy. We developed a search strategy and searched the databases till March 2018. We included individually randomised controlled trials; cluster randomised controlled trials and quasi randomised studies providing preventive or therapeutic deworming drugs for soil transmitted helminthiases and schistosomiasis during pregnancy. All IPD were assessed for completeness, compared to published reports and entered into a common data spreadsheet. Out of the seven trials elgible for IPD, we received data from three trials; out of 8,515 potential IPD participants; data were captured for 5,957 participants. Findings from this IPD suggest that mass deworming during pregnancy reduces maternal anaemia by 23% (Risk ratio [RR]: 0.77, 95% confidence intreval [CI]: 0.73–0.81; three trials; 5,216 participants; moderate quality evidence). We did not find any evidence of an effect of mass deworming during pregnancy on any of the other outcomes. There was no evidence of effect modification; however these findings should be interpreted with caution due to small sample sizes. The quality of evidence was rated as moderate for our findings. Our analyses suggest that mass deworming during pregnancy is associated with reducing anaemia with no evidence of impact on any other maternal or pregnancy outcomes. Our analyses were limited by the availability of data for the impact by subgroups and effect modification. There is also a need to support and promote open data for future IPDs.

## 1 PLAIN LANGUAGE SUMMARY

### 1.1 Mass deworming during pregnancy reduces anaemia but has no effect on other maternal or pregnancy outcomes

Pregnant women are at particular risk from soil transmitted helminthiasis (STH) – a group of diseases caused by infection with four intestinal parasites. Individual‐level data analysis with data from three studies shows that mass deworming during pregnancy reduces anaemia but has no effect on any other maternal or pregnancy outcomes.

### 1.2 What is this review about?

Soil transmitted helminthiasis (STH) are a group of diseases caused by infection with four intestinal parasites (two types of hookworm, roundworm, and whip worm) which contributed to a total of 4.98 million years lived with disability (YLDs) in 2010. Anaemia is one of the most common side effects of infection with STH or *schistosomes*, due to blood loss in the intestine or urinary tract. Women in low‐ middle‐income countries (LMICs) are especially prone since they may be pregnant or lactating for as much as half of their reproductive lives with over 50% of the pregnant women having iron‐deficiency anaemia.

This review explores whether the effect of mass deworming during pregnancy varies with individual characteristics (nutritional status, anaemia), intensity of infection (as assessed by egg count), infection status (including species of worm), socioeconomic status, sanitation environment and co‐interventions. The analysis uses individual patient data (IPD), which means that the original individual‐level data are obtained for the included studies and combined into a single data set.

### 1.3 What studies are included in this review?

Included studies have to be individually randomised controlled trials; cluster randomised controlled trials and quasi randomised studies providing preventive or therapeutic deworming drugs for STH and schistosomiasis during pregnancy.

From a total of 16 studies on mass deworming during pregnancy we identified seven trials with 8,515 participants were deemed to be eligible for individual data analysis. Of these seven trials, we received data from three trials so that out of 8,515 potential observations data were captured for 5,957.

### 1.4 What are the findings of this review?

Mass deworming during pregnancy reduces maternal anaemia by nearly one quarter (23%).

There is no effect of mass deworming during pregnancy on any of other outcomes including *Trichiura* infection, hookworm infection, low birthweight (LBW), and preterm birth.

The size of the effect is not affected by *Trichiura* intensity at baseline, maternal anaemia at baseline and maternal BMI at baseline. However these findings should be interpreted with caution due to small sample sizes. Other potential moderating characteristics could not be assessed because of lack of data.

The quality of evidence is rated as moderate for our findings. Further research on maternal baseline worm intensities and birth outcomes could change our findings.

### 1.5 What do the findings of this review mean?

The analyses suggest that mass deworming during pregnancy is associated with reducing anaemia with no effect on any other maternal or pregnancy outcomes. The analyses were limited by the availability of data for the impact by subgroups and effect modification and thus there is a need to assess mass deworming for STH and schistosomiasis during pregnancy in large scale programmatic settings along with an attempt to measure various individual and environmental factors that could potentially affect its impact. There is also a need to support and promote open data for future individual level data analysis.

## 2 EXECUTIVE SUMMARY/ABSTRACT

### 2.1 Background

Mass deworming is recommended as an effective strategy to prevent and treat soil transmitted helminthiases (STH) and schistosomiasis. However there is a great deal of heterogeneity in the existing evidence and the effectiveness of mass deworming in improving various maternal and newborn health outcomes is a current source of debate. Critical appraisal of existing studies suggests that these studies fail to account for various factors that could modify the effectiveness of deworming including nutritional status, type of infection, worm burden and concomitant interventions. Currently, it is difficult to establish whether mass deworming during pregnancy has beneficial effects under certain conditions and limited effects under others.

### 2.2 Objectives

The objective of the review is to use individual participant data (IPD) meta‐analysis to explore whether the effect of mass deworming during pregnancy varies with individual characteristics (nutritional status, anaemia), intensity of infection (as assessed by egg count), infection status (including species of worm), socioeconomic status, sanitation environment and co‐interventions.

### 2.3 Search methods

We developed a search strategy with an information scientist to search MEDLINE, CINAHL, LILACS, EMBASE, the Cochrane Library, Internet Documents in Economics Access Service (IDEAS), Google Scholar, Web of Sciences, Social Services Abstracts, WHO Global Health Library, Global Health CABI and CAB Abstracts till March 2018. We also searched grey literature, websites, contacted authors and screened references of relevant systematic reviews.

### 2.4 Selection criteria

We included individually randomised controlled trials; cluster randomised controlled trials and quasi randomised studies providing preventive or therapeutic deworming drugs for STH and schistosomiasis during pregnancy.

### 2.5 Data collection and analysis

We contacted all eligible study authors to invite them to join our investigators’ collaborative group and share their IPD. We used a data sharing agreement. All IPD were assessed for completeness, compared to published reports and entered into a common data spreadsheet. Risk of bias was assessed using the Cochrane Risk of Bias tool. Overall quality of the evidence was assessed using the Grading of Recommendations, Assessment, Development and Evaluations (GRADE) methods. This review was registered as a protocol in the Campbell Collaboration Library.

### 2.6 Results

We screened 23,406 records and identified a total of 16 studies on mass deworming during pregnancy; out of which seven trials with 8,515 participants were deemed to be eligible for IPD. Trial authors were contacted for all seven trials deemed eligible for the IPD. Out of the seven trials, we received data from three trials; data from two trails were lost (trialists were not able to retrieve the data); one trialist refused to share the data while one could not be contacted due to severe health conditions. In terms of the number of participants; out of 8,515 potential IPD participants; data was captured for 5,957 participants.

Findings from this IPD suggest that mass deworming during pregnancy reduces maternal anaemia by 23% (Risk ratio (RR): 0.77, 95% confidence intreval (CI): 0.73–0.81; three trials; 5,216 participants; moderate quality evidence). We did not find any evidence of an effect of mass deworming during pregnancy on any of other outcomes including *Trichiura* infection (RR: 0.69, 95% CI: 0.42–1.13; two trials; 2,867 participants; moderate quality evidence), hookworm infection (RR: 0.52, 95% CI: 0.18–1.47; two trials; 2,867 participants; moderate quality evidence), low birthweight (LBW) (RR: 0.89, 95% CI: 0.67–1.18; two trials; 2,267 participants; moderate quality evidence) and preterm birth (RR: 0.69, 95% CI: 0.47–1.03; two trials; 2,707 participants; moderate quality evidence). Due to limited availability of the data on the pre‐defined effect modifiers, we could only assess for effect modification by baseline *Trichiura* infection, maternal anaemia at baseline and maternal body mass index (BMI) at baseline. There was no evidence of effect modification by *Trichiura* intensity at baseline, maternal anaemia at baseline and maternal BMI at baseline. However these findings should be interpreted with caution due to small sample sizes.

The quality of evidence is rated as moderate for our findings. Further research on maternal baseline worm intensities and birth outcomes could change our findings.

### 2.7 Authors’ conclusions

Our analyses suggest that mass deworming during pregnancy is associated with reducing anaemia with no impact on any other maternal or pregnancy outcomes. Our analyses were limited by the availability of data for the impact by subgroups and effect modification and thus there is a need to assess mass deworming for STH and schistosomiasis during pregnancy in large scale programmatic settings along with an attempt to measure various individual and environmental factors that could potentially affect its impact. There is also a need to support and promote open data for future IPDs.

### 2.8 Role of the funder

The Bill and Melinda Gates Foundation had no influence on the conclusions or publication.

## 3 BACKGROUND

### 3.1 The problem, condition or issue

Soil transmitted helminthiasis (STH) are a group of diseases caused by infection with four intestinal parasites: *Ascaris lumbricoides* (roundworm), *Trichuris trichiura* (whip worm), *Necator americanus* (hookworm) and *Ancylostoma duodenale* (hookworm). Schistosomiasis is also a parasitic disease caused by blood flukes of the genus *Schistosoma*. Six species of schistosomes are responsible for infection in humans: *Schistosoma guineensis, Schistosoma haematobium, Schistosoma intercalatum, Schistosoma japonicum, Schistosoma mansoni* and *Schistosoma mekongi; S. haematobium* and *S. mansoni* are predominant causes of disease. An estimated 438.9 million people were infected with hookworm in 2010, 819.0 million with roundworms and 464.6 million with whipworm. STH altogether, contributed to a total of 4.98 million years lived with disability (YLDs) (Pullan, Smith, Jasrasaria & Brooker, 2014). Of these YLDs, 65% were attributable to hookworm, 22% to roundworm and the remaining 13% to whipworm. In terms of geographical distribution, around 67% of STH occurred in Asia contributing to 68% of the YLDs (Pullan et al., 2014). Over 270 million preschool‐age children and over 600 million school‐age children live in STH endemic areas and an estimated 4 million pregnancies a year are complicated by maternal hookworm infection alone (Bundy, Chan & Savioli, 1995; WHO, 2005).

Anaemia is one of the most common side effects of infection with STH or *schistosomes*, due to blood loss in the intestine or urinary tract. Women in low‐ middle‐income countries (LMICs) are especially prone since they may be pregnant or lactating for as much as half of their reproductive lives with over 50% of the pregnant women having iron‐deficiency anaemia. Although iron‐deficiency anaemia is multifactorial, hookworm infection is an important contributor in endemic areas, especially among women of reproductive age. An analysis on anaemia epidemiology based on data from the Global Burden of Diseases, Injuries and Risk Factors (GBD) 2010 study suggested that hookworm and Schistosomiasis were among the top ten causes of anaemia among females in 2010 (Kassebaum et al., 2014). It is the leading cause of pathological blood loss in tropical and subtropical regions (Pawlowski, Schad & Stott, 1991). Moreover there is a direct association between the intensity of STH infection, blood loss and consequent anaemia, especially for hookworms (Bundy et al., 1995; Chan, Medley, Jamison & Bundy, 1994; Larocque, Casapia, Gotuzzo & Gyorkos, 2005). The association between anaemia during pregnancy and adverse pregnancy outcomes, including low birth weight (LBW), preterm birth, perinatal mortality and infant survival has already been documented (Rahman et al., 2016; Sifakis & Pharmakides, 2000). Furthermore, the chances of favourable pregnancy outcomes are reduced by 30% to 45% in anaemic mothers, with their infants having less than one half of normal iron reserves (Rahman et al., 2016).

Mass deworming (treatment at a large scale irrespective of the diseases status) along with the water, sanitation and hygiene (WASH) interventions are generally accepted as effective measures to prevent and treat STH and Schistosomiasis. However, findings from existing studies are conflicting and the effectiveness of mass deworming in improving various maternal and child health outcomes is a current source of debate (Salam, Haider, Humayun & Bhutta, 2015; Turner et al., 2015). Critical appraisal of the existing studies suggests that these studies fail to account for various factors that could potentially modify the effectiveness of mass deworming including nutritional status, type of infection, worm burden and concomitant interventions (Barry, Simon, Mistry & Hotez, 2013; Turner et al., 2015).

### 3.2 The intervention

The World Health Organisation (WHO) recommends mass deworming (also called preventive chemotherapy, is the process of treating large numbers of people in areas with a high prevalence of these conditions) for STH and Schistosomiasis depending on prevalence of worm infection. Preventive chemotherapy (deworming), using single‐dose albendazole (400 mg) or mebendazole (500 mg), is recommended as a public health intervention for pregnant women, after the first trimester, living in areas where both:

(i) the baseline prevalence of hookworm and/or *Trichur trichiura* infection is 20% or higher among pregnant women, and
(ii) anaemia is a severe public health problem, with a prevalence of 40% or higher among pregnant women, in order to reduce the worm burden of hookworm and *T. trichiura* infection (WHO, 2017).

For Schistosomiasis, annual treatment with praziquantel in high risk communities (>50% prevalence) and once every 2 years in medium risk (>10% and <50% prevalence) is recommended and women can be treated with praziquantel at any stage of pregnancy and lactation (WHO, 2006). In addition to deworming; education on health and hygiene and provision of adequate sanitation is also recommended.

### 3.3 How the intervention might work

STH and Schistosomiasis are a major public health concern since these parasites feed on blood and affect the supply of nutrients necessary for erythropoiesis; hence contributing to anaemia (Hotez & Cerami, 1983; Torlesse & Hodges, 2000). Additionally, STH may also lead to haemorrhage by releasing anticoagulant compounds, thereby leading to iron‐deficiency anaemia. Infection during pregnancy leads to an added demand for nutrients that are critical for foetal growth and development (Abrams & Miller 2011; Blackwell, Snodgrass, Madimenos & Sugiyama, 2010). Hookworms, in particular, along with other STH and *schistosomes* have been associated with reductions in haemoglobin and iron deficiency during pregnancy (Larocque et al., 2005; Gyorkos, Gilbert, Larocque & Casapía, 2011; Muhangi et al., 2007; Nurdia, Sumarni, Suyoko, Hakim & Winkvist, 2001; Ndyomugyenyi, Kabatereine, Olsen & Magnussen, 2008bb). Additionally, STH and Schistosomiasis often occur with co‐infections in areas where malnutrition is already prevalent (Martin, Blackwell, Gurven & Kaplan, 2013).

Mass deworming is regarded as the most effective means of controlling mortality and morbidity with STH and Schistosomiasis (WHO, 2006, 2017). Preventive chemotherapy (either alone or in combination) has been used as a public heath tool for preventing morbidity due to infection usually with more than one helminth at a time since many of the antihelminthic drugs are broad spectrum. In 1994, the WHO convened an informal consultation on hookworm infection and anaemia in girls and women, which promoted the use of antihelminthics in pregnancy after the first trimester in areas where these infections are endemic and where anaemia is prevalent, but it also recommended evaluation of the long‐term safety, particularly in terms of birth outcomes (WHO, 1994). Women can be treated with praziquantel for schistosomiasis at any stage of pregnancy and during lactation. Deworming during pregnancy is often accompanied with iron supplementation to reduce anaemia.

**Figure 1:**
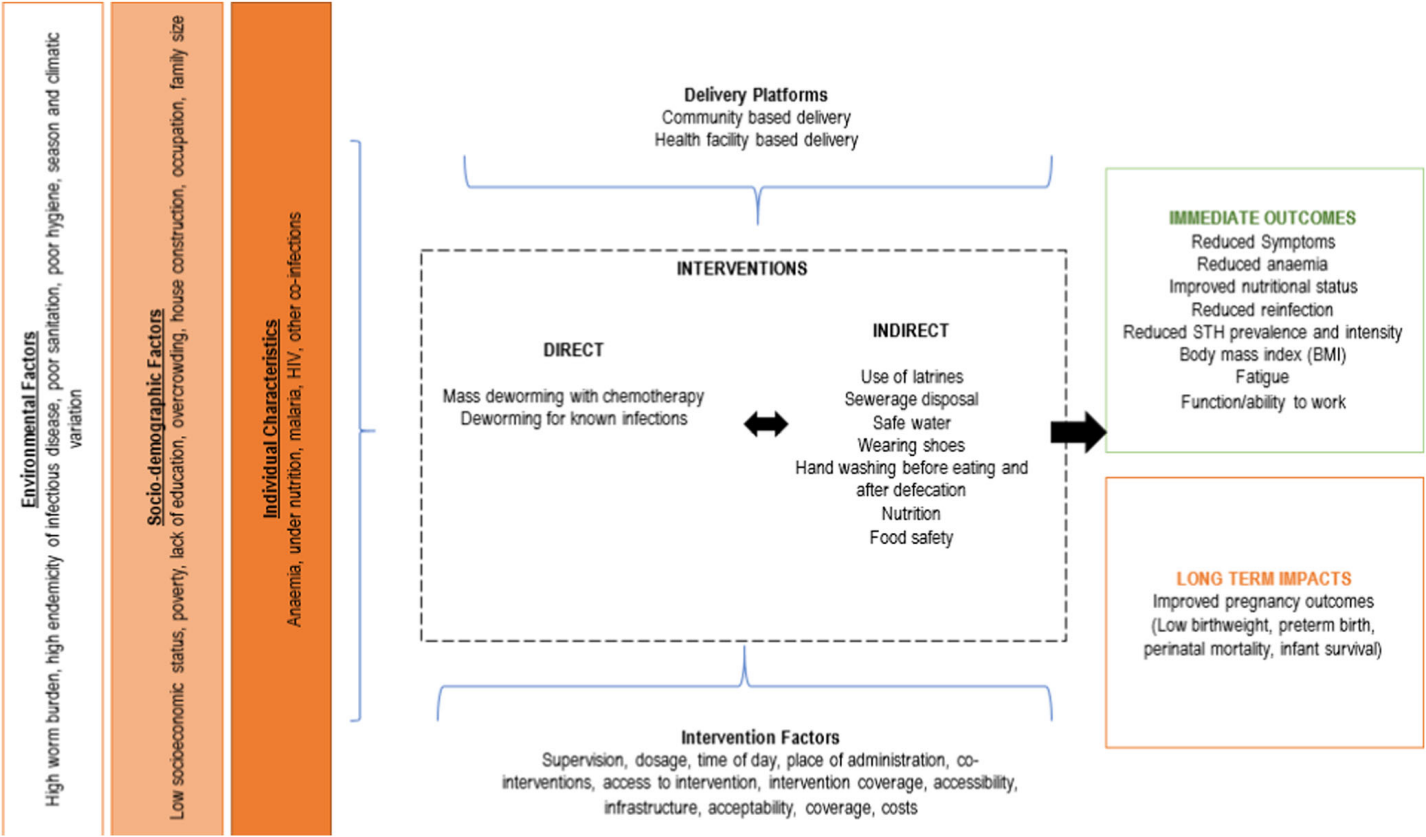
Logic model [Color figure can be viewed at wileyonlinelibrary.com]

There are various factors that could potentially modify the effectiveness of mass deworming including baseline nutritional status (anaemia and body mass index [BMI]), type of STH infection, treatment protocol, worm burden (particularly intensity of infection) and concomitant interventions (such as iron supplementation and other drugs such as praziquantel for Schistosomiasis). However, given the limited number of studies assessing the impact of deworming on maternal and newborn health outcomes (Salam et al., 2015) and complex interactions between helminthic infections and immune function, health and co‐infection risks (Blackwell, 2016), it is difficult to ascertain how these factors interplay. Currently, it is difficult to establish whether mass deworming during pregnancy has beneficial effects under certain conditions and limited effects under others and there exists a possibility that it is only beneficial in women with very high parasite burdens, dietary insufficiencies or both (Blackwell, 2016). Moreover, all intestinal worms are not the same; not all intestinal worms respond to the same deworming medication; and not all infested individuals exhibit the disease. Reinfection depends on the prevalence and intensity of infection as well as environmental factors such as the WASH practices in the community. Figure 1 highlights the logic model for this review.

### 3.4 Why it is important to do the review

A Cochrane review on deworming in the second trimester of pregnancy including four trials and 4,265 participants concluded that there was insufficient evidence to recommend deworming in pregnancy (Salam et al., 2015). There was no impact of single dose of antihelminthics administered in the second trimester of pregnancy on maternal anaemia, LBW, preterm birth and perinatal mortality. A recent Campbell systematic review and network meta‐analysis based on 47 randomised trials and over one million children, found little to no overall effect on growth, attention and school attendance (Welch et al., 2016). However, these reviews were conducted at the study level, rather than using data for each individual participant, which limits the power to detect effect modification by individual participant characteristics that could potentially modify the effect of deworming including baseline nutritional status, type of STH infection, treatment protocol, worm burden and concomitant interventions (such as iron supplementation; Barry et al., 2013; Turner et al., 2015).

IPD meta‐analysis refers to analysing data for each participant in the existing studies (Tierney, Pignon et al., 2015; Tierney, Vale et al., 2015). The term IPD refers to analysing data recorded for each participant in contrast to the aggregate study data in meta‐analysis. The advantage of an IPD analysis over aggregate meta‐analysis is that it has the potential to improve the quality of both the data and the analyses and consequently the reliability of the results (Tierney, Vale et al., 2015). Furthermore, it also provides an opportunity to re‐analyse the data for a range of other possibilities for example, investigating if the treatment effects varies by participant characteristics which is not possible with the aggregate data (Riley, Lambert & Abo‐Zaid, 2010). An IPD approach will allow an evaluation of variation in effect estimates by various individual, socio‐demographic and environmental factors among pregnant women that could potentially modify the effectiveness of mass deworming during pregnancy.

Despite the availability of more recent global estimates on the burden and interventions for STH and Schistosomiasis, additional research is needed to understand the factors that explain the variation in the effect estimates of recommended interventions to prevent transmission. Existing studies fail to account for various factors that could modify the effectiveness of mass deworming including underlying host and environment factors. IPD meta‐analysis would explore the question of whether mass deworming during pregnancy is more effective for subgroups of women defined by characteristics such as nutrition status and infection intensity. This understanding could help develop targeted strategies to reach pregnant women with deworming and guide policy regarding mass deworming. A companion review using IPD and network meta‐analysis to explore whether the effects of different types and frequency of deworming drugs as well as their combination with food or micronutrients vary with child‐level and study‐level characteristics is also registerd with Campbell Collaboration (Welch et al.).

## 4 Objectives

The objective of the review is to use IPD meta‐analysis to explore whether the effect of mass deworming among pregnant women on maternal and birth outcomes vary with individual characteristics (nutritional status, anaemia), intensity of infection (as assessed by egg count), infection status (including species of worm), socioeconomic status, sanitation environment and other co‐interventions.

## 5 METHODOLOGY

The protocol was registered with the Campbell Collaboration (Salam et al.) and reported according to the preferred reporting items for systematic reviews and meta‐analyses for protocols (PRISMA‐P) (Moher et al., 2015). Results of the review are reported using the Preferred Reporting items for Systematic Reviews and Meta‐analyses of individual patient data (PRISMA‐IPD) Statement (Stewart et al., 2015).

### 5.1 Criteria for including and excluding studies

We included studies that met the following eligibility criteria

#### 5.1.1 Types of study designs

We included individually randomised controlled trials (RCT); cluster RCTs and quasi randomised studies (studies where non‐random assignment is determined by factors that are out of the control of the investigator) as these were the most appropriate design for the IPD meta‐analysis. No language or date restrictions were applied.

#### 5.1.2 Types of participants

Participants were pregnant women receiving preventive or therapeutic deworming drugs for STH and schistosomiasis.

#### 5.1.3 Types of interventions

We included mass deworming using any drug or a combination of drugs (including levamisole, mebendazole, albendazole, praziquantel and pyrantel) for STH and schistosomiasis with or without co‐interventions compared to placebo or control (no mass deworming). Co‐interventions could be food provision, micronutrient supplementation, iron and/or folic acid supplementation, hygiene interventions or education. We included studies where the co‐interventions were similar in the intervention and control groups to assess the impact of mass deworming.

#### 5.1.4 Types of outcome measures

Following primary and secondary outcomes were reported; however we did not use the list of outcomes as a criteria for inclusion of studies in the review:

Primary outcomes:

- Maternal anaemia at term (haemoglobin less than 11 g/dl)
- Maternal infection intensity (as reported by the study authors)

Secondary outcomes:

- Maternal haemoglobin at term
- Maternal ferritin
- Maternal anthropometric measures (height and weight)
- Maternal body mass index (BMI)
- Birth weight
- LBW (less than 2500 g)
- Preterm birth (birth before 37 weeks of gestation)
- Perinatal mortality (includes foetal death after 28 weeks of gestation and infant death that occurs at less than seven days of life)
- Stillbirth
- Congenital abnormalities
- Infant mortality

#### 5.1.5 Duration of follow‐up

We did not restrict inclusion based on the duration of follow‐up.

#### 5.1.6 Types of settings

The settings included any area where STH or schistosomes are endemic. These could include studies conducted in either community settings or facility settings including hospitals, antenatal clinics, primary healthcare centres etc.

### 5.2 Search strategy

We conducted the search in the following databases till 21 March 2018: MEDLINE, CINAHL, LILACS, EMBASE, the Cochrane Library, Internet Documents in Economics Access Service (IDEAS), Google Scholar, Web of Sciences, Social Services Abstracts, WHO Global Health Library, Global Health CABI and CAB Abstracts. We also searched grey literature in OpenGrey and websites of relevant organisations such as the World Bank, World Food Program and International Food Policy Research Institute. We also contacted authors of studies and members of our advisory board for any unpublished studies or grey literature reporting eligible studies. We checked reference lists of relevant studies and reviews. We also searched for trials registered with ClinicalTrials.gov and the WHO International Clinical Trials Registry Platform (http://www.who.int/trialsearch/).

Titles and abstracts were screened in duplicate by two reviewers. We pilot‐tested the screening criteria at both title and abstract screening stage and full text stage. We used the PRISMA flow diagram to report eligibility of studies. We retrieved full text of all studies which pass this first level screening. The full text review were also done in duplicate by two reviewers, and agreement was reached by consensus. Disagreements were resolved by consultation with a third reviewer. No language or date limits were applied. The search strategy is attached as Appendix 1.

### 5.3 Description of methods used in primary research

RCTs of mass deworming include two‐arm trials as well as factorial trials, with women allocated either individually or by cluster‐randomisation.

### 5.4 Details of study coding categories

We extracted the study characteristics including details of the populations, setting, socio‐demographic characteristics, interventions, comparators, outcomes and study design in duplicate. The characteristics extracted from the included studies are specified in Appendix 2.

### 5.5 Quality assessment and grading

Risk of bias was assessed at the study as well as the outcome level. At the study level, two independent reviewers performed quality appraisal for each study using the Cochrane risk of bias tool which assessed selection bias, performance bias, detection bias, attrition bias and reporting bias (Higgins, Altman & Sterne, 2011). Disagreements were resolved by discussion or consultation with a third reviewer. At the outcome level, we summarised the quality of evidence according to the outcomes as per the Grading of Recommendations Assessment, Development and Evaluation (GRADE) criteria (Walker et al., 2010). A grade of 'high', 'moderate', ‘low’ and ‘very low’ was used for grading the overall evidence indicating the strength of an effect on specific health outcome based on methodological flaws within the component studies, consistency of results across different studies, generalisability of research results to the wider patient base and how effective the treatments have shown to be (Balshem et al., 2011). The two reviewers discussed ratings and reached consensus. Disagreements were resolved by consulting a third reviewer. We developed a summary of findings table to show the effects for the primary outcomes of maternal anaemia and infection intensity; as well as the secondary outcomes of preterm birth, LBW and perinatal mortality since these outcomes assess long‐term effects, particularly in terms of birth outcomes.

### 5.6 Statistical procedures and conventions

Trialists of the included trials provided IPD by electronic transfer where possible or other means as needed. The individual trial data were recoded as required and checked with respect to range, internal consistency, missing values, outliers, errors and consistency with published reports. Trial details such as randomisation methods and intervention details were cross‐checked against published reports, trial protocols and data collection sheets. Inconsistencies or missing data were discussed with the individual trialists and attempts were made to resolve any problems by consensus. We did not exclude any study based on the way the outcomes were reported.

Data were prepared into a flat spread‐sheet with the same fields for every study. We considered the missing values for each variable as missing at random (MAR). For this IPD, we restricted our analysis to conventional complete case analyses, that is, removing subjects with a missing value from the analyses, since the missing data were considered to be non‐trivial. For future updates, we plan to use multiple imputation to impute the missing values for covariates at baseline (individual participant level variables) and outcome variables (primary and secondary outcomes). Imputation was planned to be done using Proc MI in SAS/STAT (SAS Institute Inc., Cary, NC). We plan to assess the robustness of the results by running a separate model excluding imputed data (i.e., complete case analysis). We plan to include studies with missing data on more than 50% of outcome or covariate data in the complete case analysis only for future updates.

Descriptive characteristics of each study were presented, with details on the participant characteristics, environment, worm species, prevalence, intensity of infection, geographic location, interventions, comparator, outcomes and risk of bias assessment. We accounted for clusters (such as villages, schools or households) as nested within each study. Following data items were collected:

Individual level:

- Infection intensity with *Ascaris, Trichuris*, hookworm and schistosomes (across four levels of none, light, moderate and heavy, using the WHO cutoffs for each helminth, available at: http://apps.who.int/iris/bitstream/10665/44671/1/9789241548267_eng.pdf)
- Anaemia status (using WHO cutoffs by age and altitude of non‐anaemic, mild, moderate and severe, http://www.who.int/vmnis/indicators/haemoglobin.pdf)
- Undernutrition (BMI < 18.5 kg/m^2^)
- Socioeconomic status (as defined by trial authors): We assessed whether the measurement of socioeconomic status can be compared across study settings and time.
- Deworming drug used.

Environmental level:

- WASH practices (as defined by trial authors)
- Population level infection intensity (using WHO cut‐offs for each worm‐type, as above)

We calculated the standardised difference between the published data and the IPD received from authors for baseline characteristics and baseline outcome assessment. For endline (outcome measures), we replicated the effect measures reported in study publications and calculated the standardised difference between the IPD received and the study report (Austin, 2009).

The comparison of interest for the pairwise analysis included (but not restricted to) any deworming drug versus no deworming. We used a two‐step process to meta‐analysis. We conducted pairwise analyses for the comparison of interest by entering all IPD data into a multilevel model, with each study as one cluster. We expected considerable heterogeneity between studies for each outcome; therefore, we used a random effects model. Where IPD was not available for all trials, we used a two‐part model with one part based on IPD data and the second part based on aggregate data from studies which did not provide IPD (Fisher, Copas, Tierney & Parmar, 2011; Riley & Steyerberg, 2010; Riley et al., 2008). We planned to conduct pair‐wise comaprisons for one deworming drug versus other deworming drug or a combination of deworming drugs, however we could not perform such analysis due to limited data.

We accounted for clustering as above by nesting clusters within studies. We decided on a set of pre‐defined covariates with advice from our advisory board and co‐authors. We accounted for the pre‐defined covariates of infection intensity, baseline anaemia, baseline nutritional status, socioeconomic status and maternal education in the model. We did not plan to conduct network meta‐analysis based on our previous experience with limited number of studies in the domain (Salam et al., 2015).

### 5.7 Measures of treatment effects

We separately analysed the dichotomous and continuous outcomes. For dichotomous outcomes, we presented the results as summary risk ratios (RRs) with 95% confidence intervals (CI). We presented continuous outcome data as either a mean difference (MD), if outcomes have been measured on the same scale, or a standardised mean difference (SMD), if outcomes have been measured on different scales, with 95% CI. For each outcome, we reported the results for the evidence from study results pooled at the aggregate level (adjusted for covariates) and the evidence pooled using IPD (adjusted for covariates).

### 5.8 Assessment of clinical and methodological heterogeneity within treatment comparisons

Heterogeneity across trials in terms of subject characteristics, trial methodologies and treatment protocols was assessed using visual plots, tables and homogeneity statistics. We assessed heterogeneity using visual inspection of forest plots for pairwise analyses as well as statistical tests of heterogeneity (*I* ^2^). In addition to *I* ^2^, we also assessed between‐study variance (variation across study findings beyond random sampling error) by the variance of the distribution of the true study effects, commonly denoted as *τ* ^2^.

### 5.9 Publication bias

We planned to generate a funnel plot for comparisons and outcomes with >10 studies. We planned to use Egger's test for asymmetry and visual inspection to assess the presence of publication bias and/or selective reporting. However, none of the comparisons or outcomes included >10 studies and hence we could not assess for publication bias.

### 5.10 Subgroup analyses

Where sufficient data were available, sub‐group analyses was planned to be conducted to assess effects across both individual‐level as well as environment‐level characteristics. We compared the results of models with subgroup analyses by assessing the size of quantitative or qualitative differences in effects, the statistical significance of tests for interactions, assessing between‐study variance and assessing the goodness of fit of the models using the likelihood ratio. Before conducting subgroup analyses, we assessed the distribution of each variable. If there were insufficient participants in some categories, the levels were combined. The following individual and environment level effect modifiers were planned to be assessed (data permitting):

Individual Level:

- Infection intensity with *Ascaris, Trichuris*, hookworm and schistosomes (across four levels of none, light, moderate and heavy, using the WHO cutoffs for each helminth, available at: http://apps.who.int/iris/bitstream/10665/44671/1/9789241548267_eng.pdf)
- Anaemia status (using WHO cutoffs by age and altitude of non‐anaemic, mild, moderate and severe, http://www.who.int/vmnis/indicators/haemoglobin.pdf)
- Undernutrition (BMI < 18.5 kg/m^2^)
- Socioeconomic status (as defined by trial authors): We assessed whether the measurement of socioeconomic status can be compared across study settings and time.

Environmental Level:

- WASH practices (as defined by trial authors)
- Population level infection intensity (using WHO cut‐offs for each worm‐type, as above)

### 5.11 Sensitivity analyses

Where sufficient data were available, we planed to conduct sensitivity analyses to assess robustness of results when restricted to studies at low risk of bias for sequence generation, allocation concealment and blinding of participants. We planned to assess whether results were robust to excluding imputed data (i.e., complete case analysis).

### 5.12 Data management

Data were transferred to SAS as a common platform for all studies, using a common data dictionary. We checked IPD data for consistency immediately upon receiving datasets for outlier individuals (e.g. with duplicate participant IDs, unrealistic date ranges). We compared the IPD from authors with the aggregate data reported in the articles. Any missing or unusual data were flagged for discussion with the trial author or statistician. We asked for clarification from the authors to establish reasons for the errors, and correct them if possible. Any requests for authors were discussed when the data were provided, such as clarification of trial risk of bias, conduct or eligibility criteria. We also ran the same statistical analysis as the authors to check for consistency with the published paper (Stewart et al., 2015). We requested statements of ethics approval from each study and we did not include data from studies that did not receive ethics approval. We requested that all data be transferred without any identifiers.

### 5.13 Treatment of qualitative research

We did not plan to include qualitative research.

## 6 RESULTS

The results of this review are reported according to the PRISMA‐IPD reporting guidelines (checklists in Additionals Table 1).

### 6.1 Search results

**Figure 2:**
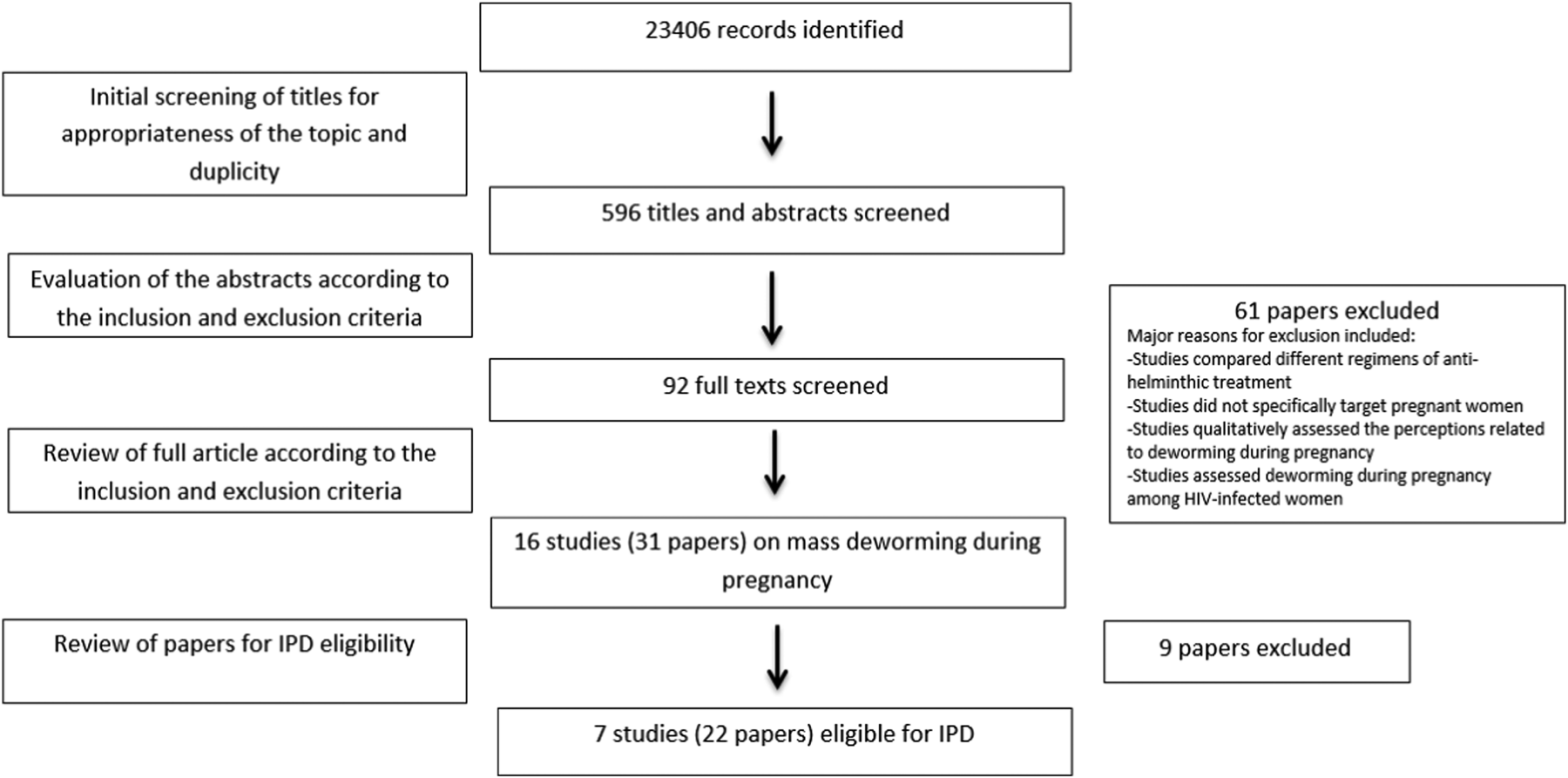
Search flow diagram

We searched all databases up to March, 2018. Figure 2 provides a search flow diagram. We identified a total of 23406 record through the search strategy provided in Appendix 1. A total of 31 papers (Atukorala, de Silva, Dechering, Dassenaeike & Perera, 1994; Villar et al., 1998; Abel, Rajaratnam, Kalaimani & Kirubakaran, 2000; Ács, Banhidy, Puho & Czeizel, 2005; Adam, Elwasila & Homeida, 2005; Christian, Khatry & West, 2004; De Silva, Sirisena, Gunasekera, Ismail & de Silva, 1999; Deepti & Nandini, 2015; Elliott, Mpairwe et al., 2005; Elliott, Namujju et al., 2005; Elliott et al., 2007; Gyorkos, Larocque, Casapia & Gotuzzo, 2006; Gyorkos, Gilbert et al., 2011; Larocque et al., 2006; Liabsuetrakul et al., 2009; Tehalia, 2011; Mpairwe et al., 2011; Millard et al., 2014; Nampijja et al., 2012; Ndibazza et al., 2010; Ndibazza et al., 2012; Ndyomugyenyi, Kabatereine, Olsen & Magnussen, 2008aa; Olveda et al., 2016; Torlesse & Hodges, 2000; Torlesse & Hodges, 2001; Tweyongyere et al., 2008; Tweyongyere et al., 2009; Tweyongyere et al., 2011; Tweyongyere et al., 2013; Urassa, Nystrom & Carlsted, 2011; Webb, Mawa et al., 2011; Webb, Kyosiimire‐Lugemwa et al., 2012) based on 16 studies assessed mass deworming during pregnancy and were deemed eligible for the review. These 16 studies were assessed for IPD eligibility and seven studies with 8,515 participants were identified to be eligible for IPD. Major reasons for exclusion from IPD inlcuded:

(i) study design not being appropriate and;
(ii) only abstracts were available with insufficient information and the trialists could not be contacted.

Out of the seven studies found eligible for IPD, three trials were subsequently included in the IPD since the authors of these three trials provided data for IPD (Elliott, Mpairwe et al., 2005; Olvedaet al., 2016; Urass et al., 2011). Table 1 details the study eligibility for IPD.

**Table 1:** Eligibility for IPD

Study ID	Study Design	Eligible for IPD	Reason for Exclusion
Elliott 2005 (Ndibazza et al., 2010)	Randomised Controlled Trial	Yes	
Larocque 2006 (Larocque et al., 2006)	Randomised Controlled Trial	Yes	
Ndyomugyenyi 2008 (Ndyomugyenyi et al., 2008aa)	Randomised Controlled Trial	Yes	
Torlesse 2001 (Torlesse & Hodges, 2001)	Randomised Controlled Trial	Yes	
Urassa 2011 (Urass et al., 2011)	Randomised Controlled Trial	Yes	
Deepti 2015 (Deepti & Nandini, 2015)	Randomised Controlled Trial	Yes	
Tehalia 2011 (Tehalia, 2011)	Randomised Controlled Trial		Only abstract available with insufficient information and the authors could not be contacted
Villar 1998 (Villar et al., 1998)	Randomised Controlled Trial		Only abstract available with insufficient information and the authors could not be contacted
Olveda 2016 (Olvedaet al., 2016)	Randomised Controlled Trial	Yes	
Atukorala 1994 (Atukorala et al., 1994)	Before‐after study		Study design not appropriate
Abel 2000 (Abel et al., 2000)	Before‐after study		Study design not appropriate
Christian 2004 (Christian et al., 2004)	Prospective Cohort		Study design not appropriate
de Silva 1999 (De Silva et al., 1999)	Cross‐sectional survey		Study design not appropriate
ACS 2005 (Ács et al., 2005)	Case‐control study		Study design not appropriate
Adam 2005 (Adam et al., 2005)	Prospective cohort		Study design not appropriate
Liabsuetrakul 2009 (Liabsuetrakul et al., 2009)	Prospective cohort		Study design not appropriate

Abbreviation: IPD, individual participant data.

### 6.2 Characteristics of studies

A total of seven studies including 8,515 pregnant women were eligible for IPD. All of these studies were RCTs. Studies were conducted in India, Philippines, Peru, Sierra Leone, Tanzania and Uganda between 2001 and 2016. The deworming drugs provided in these studies included albendazole, mebendazole, praziquantel, ivermectin or a combination of these. Majority of the studies provided mass deworming for STH only; while one study (Olveda et al., 2016) provided deworming for schistosomiasis alone; and one study (Elliott, Mpairwe et al., 2005) targeted both STH and schistosomiasis. The sample size ranged from 184 pregnant women to 3,080 pregnant women. The most common co‐intervention was iron/folic acid supplementation while other interventions included food supplementation, anti‐malarial drug administration and education. Maternal and birth outcomes were assessed in the third trimester and at the time of delivery in all the included studies. Table 2 describes the characteristics of studies eligible for IPD. Out of the seven studies, three trials were subsequently included in the IPD (Elliott, Mpairwe et al., 2005; Olveda et al., 2016; Urass et al., 2011) and further descritipion is provided in the following sections.

**Table 2:** Characteristics of IPD eligible studies

Serial no	Study ID	Study design	Country/Setting	Sample size	Intervention	Control group	Baseline characteristics reported	Outcomes reported
	Elliott 2005	Randomised Controlled Trial	Entebbe Hospital, Uganda between June and August, 2002.	2507 participants	– Albendazole (400 mg) and placebo, – Praziquantel (40 mg/kg) and placebo – Albendazole and praziquantel	Placebo and placebo	Maternal education	Infection
								Infantile eczema
							Household economic index	Immune responses in mothers and infants
							Trimester at treatment	
							Parity	Maternal and perinatal outcomes
							Place of delivery	
							HIV status	Immune responses (BCG, tetanus, pertussis, hep B, measels, diptheria, polio, haemophilius)
							Malaria parasites	Co‐infections (malaria, pneumonia, diarrhoea, TB, measels, HIV)
							Active syphilis	Anaemia (haemoglobin concentration)
							Helminth prevalence (Hookworm, schisto, *Trichuris*, Ascaris lumbricoides)	Growth and development (birth weight, weights, height, head circumference, MUAC, intellectual function)
								Worm infection
							Anaemia	Mortality
	Larocque 2006	Randomised Controlled Trial	Health centres in the Iquitos region of Peru	1042 participants	Single dose of mebendazole (500 mg) plus a daily iron supplement (60 mg elemental iron, ferrous sulphate)	Single dose placebo plus a daily iron supplement (60 mg elemental iron, ferrous sulphate)	Gestational age	Mean infant birthweight (LBW and VLBW)
							Environment (Urban/rural)	
							Schooling	Maternal anaemia in third trimester measured by (a) mean Hb and (b) Hb < 11 g/dL
							Primigravida	
							Housing	
							Flooring	
							Toilet facility	Infection prevalence
							Water use	Stillbirth
							STH prevalence (hookworm, *Trichuris*, ascaris, coinfection with hookworm/*Trichuris*)	Early neonatal death
								Term birth
							STH intensities	Miscarriage
							Anaemia	Malformations
							Haemoglobin	
	Ndyomugyenyi 2008a	Randomised Controlled Trial	Masindi district, western Uganda	832 participants	Group A (n = 198) received ivermectin	Group D (n = 241) was a reference group without soil‐transmitted helminths.	Weight	Maternal Hb in third trimester
							Height	
					Group B (n = 194) received albendazole (a single dose of 400 mg)		Hb	Birthweight
							Gestational age	LBW
								Abortion
								Stillbirths
					Group C (n = 199) received a combination of ivermectin and albendazole, and Women in addition received the routine antenatal care package with iron supplements			Neonatal death
								Preterm birth
								Cure rate
								Mean parasite density
								Neonatal anaemia
								Neonatal mean Hb
	Torlesse 2001	Randomised Controlled Trial	Antenatal clinics in peri‐urban and 6 in rural areas in Sierra Leone	184 participants	Albendazole, 2 × 200 mg, single dose, at first antenatal visit in second trimester. Daily iron‐folate supplements comprised 36 mg iron	Two tablets containing calcium with vitamin D were used as the control for albendazole. calciferol tablets (1.25 as ferrous gluconate and 5 mg folic acid started at first antenatal visit in second trimester for entire duration of pregnancy. mg), 1 daily, were chosen as the control for iron‐folate supplements	Hb	Worm prevalence
								Anaemia
								Iron deficiency anaemia
								Cure rate
								Egg reduction rate
	Urassa 2011	Randomised Controlled Trial	Rufiji district, Tanzania	3080 participants	Single dose Albendazole (400 mg) (given at term and 4 months later)	Placebo	Parity	Haemoglobin
							Gestational age	Serum ferritin concentration during pregnancy
					Daily iron folate supplements (36 mg iron; 5 mg folate)		Distance of facility from residence	
							Knowledge of anaemia	
					Sulphadoxine pyramethamine		Knowledge of malaria	Anaemia
							Hb	
							Anaemia	
	Deepti 2015	Randomised Controlled Trial	India	500 participants	– Albendazole – Mebendazole – Albendazole and mebendazole	Placebo	Education	Maternal anaemia
							Socio‐economic status	Worm intensity
							Hb	Worm prevalence
							Baseline infestation	Birth weight
								Low birth weight
	Olveda 2016	Randomised Controlled Trial	Villages in northeastern Leyte, Philippines	370 pregnant women	over‐encapsulated praziquantel (total dose 60 mg/kg given	Placebo	Socio‐economic status	Birth weight
							Height	LBW
					as two split doses)		Weight	SGA
							Baseline prevalence	Maternal Hb
								Newborn Hb
								Maternal weight gain
								Tretament success
								Cure rate
								Maternal adverse events
								Congenital anomaly
								Foetal death
								Abortion

## 7 QUALITY OF STUDIES

**Figure 3:**
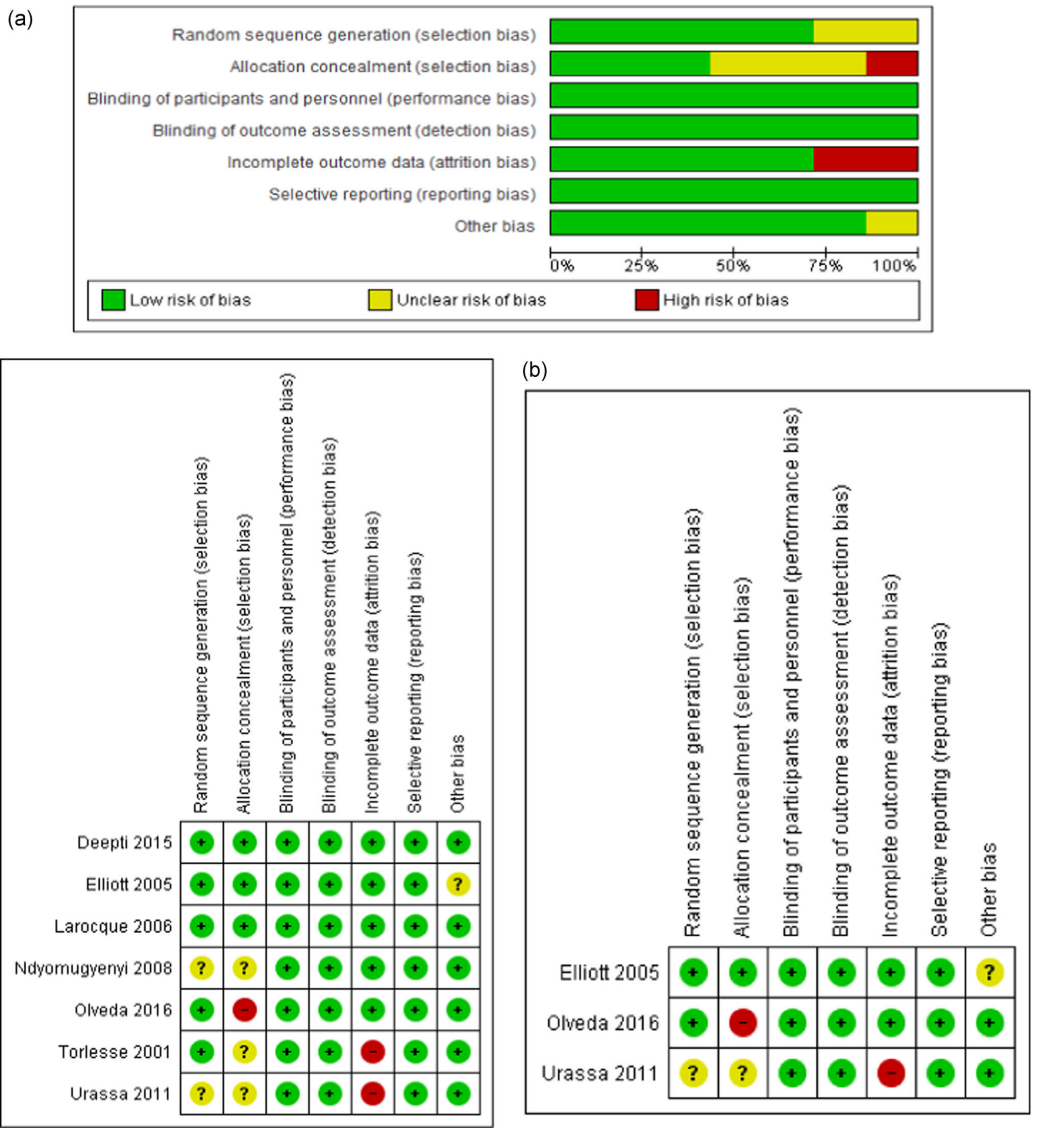
(a) Risk of bias for the included trials. (b) Risk of bias for the trials included in IPD. IPD, individual participant data [Color figure can be viewed at wileyonlinelibrary.com]

The quality of the studies was assessed using the Cochrane risk of bias assessment criteria. Overall, the included studies were judged to be of fairly good quality. For random sequence generation, five studies were judged to be at low risk of bias while two studies (Ndyomugyenyi et al., 2008aa; Urass et al., 2011) were rated as unclear since the method of sequence generation was not specified. Allocation concealment was judged to be adequately done in three studies (Larocque et al., 2005; Deepti & Nandini, 2015; Elliott, Mpairwe et al., 2005); three studies did not clearly specify the concealment of allocation and were judged to be at unclear risk (Torlesse & Hodges, 2000; Ndyomugyenyi et al., 2008aa; Urass et al., 2011) while one study did not adequately conceal the allocation and was rated as high risk for allocation concealment (Olveda et al., 2016). All the included studies either adequately blinded the participants, personnels and outcome assessors or we felt that lack of blinding would be unlikely to affect the results and hence all the studies were rated to be a low risk for blinding. Four studies were rated at low risk of attrition bias while two studies were rated to be at high risk of attrition bias (Torlesse & Hodges, 2000; Urass et al., 2011). All the studies were judged to be at low risk of bias for selective reporting since the outcomes specified in the study protocol or methodology section of the study were reprotred in the outcome section. We judged one study as unclear risk of bias for ‘other bias’ since in the (Elliott, Mpairwe et al., 2005) study, enrolment was stopped after 104 women due to new guidelines by the WHO which recommended inclusion of treatment of women with schistosomiasis. Figure 3a depicts the risk of bias for the studies included in the review while Figure 3b depicts the risk of bias for the studies subsequently included in the IPD.

## 8 CONTACTING AUTHORS AND YIELD OF THE STUDIES

**Figure 4:**
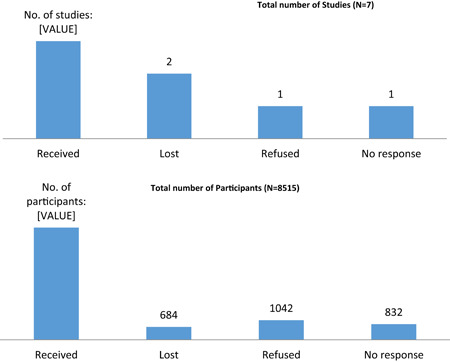
Number of eligible studies and participants for IPD. IPD, individual participant data [Color figure can be viewed at wileyonlinelibrary.com]

Trial authors were contacted for all seven trials deemed eligible for the IPD. Out of the seven trials, we received data from three trials (Elliott, Mpairwe et al., 2005; Olveda et al., 2016; Urass et al., 2011); data from two trails were lost (Deepti & Nandini, 2015; Torlesse & Hodges, 2000; trialists were not able to retrieve the data); one trialist refused to share the data (Larocque et al., 2006) while one could not be contacted due to severe health conditions (Ndyomugyenyi et al., 2008aa). In terms of the number of participants; out of 8,515 potential IPD participants; data were captured for 5,957 participants. Figure 4 depicts the number of studies and participants eligibility for IPD.

## 9 DATA AND ANALYSIS

### 9.1 Data preparation: missingness analysis

Tables 3 and 4 provides an overview of the missing values for the baseline and endline variables in the data sets from each of the trial.

**Table 3:** Missing values for baseline variables

	Studies			
Baseline variables	Elliott, 2005	Olveda, 2016	Urassa, 2011	Total *N* = 5,943
	(*n* = 2,505)	(*n* = 362)	(*n* = 3,076)	
Education	0.15% (4)	0.55% (2)	NA	0.1% (6)
Parity	NA	0%	0%	0%
Gravidity	0%	NA	0%	0%
Weight	0.15% (5)	0%	NA	0.08% (5)
Height	1.12% (28)	0%	NA	0.5% (28)
Anaemia	0.5% (12)	0%	0%	0.2% (12)
*S.Japonicum* intensity	NA	0%	NA	0%
*S.Mansoni* intensity	0%	NA	NA	0%
*A.Lumbricoides* intensity	NA	39% (141)	NA	2.4% (141)
*T.Trichiura* intensity	0%	19% (69)	NA	1.15% (69)
Hookworm intensity	0%	64% (231)	NA	3.88% (231)
Ascaris intensity	0%	NA	NA	0%
Socioeconomic status	7.3% (183)	0%	NA	3.08% (183)

**Table 4:** Missing values for endline variables

	Studies			
Endline variables	Elliott, 2005	Olveda, 2016	Urassa, 2011	Total*N* = 5,943
	(*n *= 2,505)	(*n* = 362)	(*n* = 3,076)	
Weight	NA	0.82% (3)	NA	0.05% (3)
Anaemia	13.53% (339)	0.82% (3)	12.51% (385)	12.23% (727)
*S.Japonicum* intensity	NA	63.5% (230)	NA	3.87% (230)
*S.Mansoni* intensity	18% (451)	NA	NA	18% (451)
*A.Lumbricoides* intensity	NA	0%	NA	0%
*T.Trichiura* intensity	18% (451)	26% (94)	NA	9.17% (545)
Hookworm intensity	18% (451)	0%	NA	18% (451)
Ascaris intensity	18% (451)	0%	NA	18% (451)
Birth weight	23.91% (599)	0.27% (1)	NA	10.1% (600)
LBW	23.91% (599)	0.27% (1)	NA	10.1% (600)
Preterm birth	6.38% (160)	0%	NA	2.7% (160)

### 9.2 Data replications

Replication of the published study results was conducted for all three studies. The standardised differences between the published and replication results were all below 0.10 for all outcome measures and covariates. There were instances where the standardised difference could not be calculated because the published results did not report the outcome measure in question. Table 5 reports the standardised differences between the published and reproduced results for outcome measures.

**Table 5:** Standardised differences between published and reproduced results for outcome measures by eligible studies

	Studies		
Variables	Elliott, 2005	Olveda, 2016	Urassa, 2011
Maternal weight	NA	0.00	NA
Maternal anaemia	0.02	NA	0.00
Maternal haemoglobin	0.04	0.005	NA
*S. Japonicum* intensity	NA	NA	NA
*S. Mansoni* intensity	0.00	NA	NA
*Ascaris* intensity	0.00	NA	NA
*Trichuris* intensity	0.04	NA	NA
Hookworm intensity	0.00	NA	NA
Birth weight	0.007	0.003	NA
LBW	0.05	0.05	NA
SGA	NA	0.00	NA
Preterm birth	NA	NA	NA
Perinatal mortality	0.00	NA	NA
Congenital anomaly	0.01	NA	NA
Infant survival	NA	NA	NA

## 10 IPD FEASIBILITY AND CHANGES TO THE ANALYSIS MODEL

Based on the availability of data, we could only analyse one comparison of interest (mass deworming with any drug versus no mass deworming). The planned analysis and final model was also modified accordingly. Table 6 provides a comparison of the original analysis plan and the actual analysis model.

**Table 6:** Comparison of the original analysis plan and actual model employed

	Planned analysis	Actual analysis
Outcomes	Maternal anaemia at term	Maternal anaemia at term (Hb < 109 g/l)
	Maternal infection intensity	*Trichiura* intensity (none vs. any (light/moderate/heavy) intensity)
	Maternal haemoglobin at term	Hookworm intensity (none vs. any (light/moderate/heavy) intensity)
	Maternal ferritin	
	Maternal anthropometric measures	LBW (<2500 g)
		Preterm birth (<37 weeks of gestation)
	Maternal BMI	
	Birth weight	
	Low birth weight	
	Preterm birth	
	Perinatal mortality	
	Stillbirth	
	Congenital abnormalities	
	Infant Mortality	
Covariates	*Schistosoma* intensity	Hookworm intensity (none vs. any (light/moderate/heavy) intensity)
	*Ascaris* intensity	*Trichiura* intensity (none vs. any (light/moderate/heavy) intensity)
	Hookworm intensity	Haemoglobin (Hb < 109 g/l)
	*Trichuria* intensity	
	Haemoglobin	
	BMI	
	Socio‐economic status	
	Deworming drug	
	WASH practices	
	Population level worm intensities	
Effect Modifiers	BMI (<18.5 kg/m^2^, 18.5 to 25 kg/m^2^)	BMI (<18.5 kg/m^2^, 18.5 to 25 kg/m^2^)
	Anaemia status (none, mild, moderate, severe)	Aanemia (Hb<109 g/l)
	*Schistosoma* intensity (light, moderate, heavy)	*Trichiura* intensity (none vs. any (light/moderate/heavy) intensity)
	*Ascaris* intensity (light, moderate, heavy)	
	Hookworm intensity (light, moderate, heavy)	
	*Trichuria* intensity (light, moderate, heavy)	
	Any STH or *Schistosoma* infection (light, moderate, heavy)	
	Concomitant interventions	

## 11 MAIN EFFECTS

This section provides the overall results for mass deworming compared to no mass deworming on the following outcomes: maternal anaemia; maternal infection intensity (*T.Trichiura* and hookworm); LBW and preterm birth. All of the seven trials deemed eligible for the IPD contributed data towards the aggregate estimate while data from three trials (Elliott, Mpairwe et al., 2005; Olveda et al., 2016; Urass et al., 2011) contributed to the IPD estimate. We report results for the evidence from study results pooled at the aggregate level (adjusted for covariates) and the evidence pooled using IPD (adjusted for covariates). However we advise caution in interpreting these findings due to small sample sizes.

Following this section, we describe effect modifier analyses for each planned effect modifier for each outcome of interest.

### 11.1 Maternal anaemia

The effect estimates from aggregate evidence were of similar size and direction as the IPD effect estimates. Three trials reported data on maternal anaemia. Mass deworming led to a 23% reduction in maternal anaemia (RR: 0.77, 95% CI: 0.73–0.81; three trials; 5,216 participants; moderate quality evidence). Table 7 reports the aggregate and IPD adjusted estimates.

**Table 7:** Imapct of mass deworming on maternal anaemia

Analysis	Effect estimates (RR and 95% CI)
Aggregate adjusted	0.94 (0.89–0.99)
IPD adjusted	0.77 (0.73‐0.81)

### 11.2 *T.Trichiura* intensity

Two trials reported *T.Trichiura* intensity showing no evidence of impact of mass deworming on any infection (RR: 0.69, 95% CI: 0.42–1.13; two trials; 2,867 participants; moderate quality evidence). We attempted to categorise the participants according to the intensity of infection (none, light, moderate and heavy); however there were too few participants in each category to draw meaningful conclusions. The effect estimates from aggregate evidence were of similar size and direction as the IPD effect estimates. Table 8 reports the aggregate and IPD adjusted estimates for maternal *T.Trichiura* intensity.

**Table 8:** Mass deworming on *T.Trichiura* intensity (any infection)

Analysis	Effect estimates (RR and 95% CI)
Aggregate adjusted	1.06 (0.87, 1.30)
IPD adjusted	0.69 (0.42–1.13)

### 11.3 Hookworm intensity

Two trials reported hookworm intensity. Overall there was no evidence of impact of mass deworming on any hookworm infection (RR: 0.52, 95% CI: 0.18, 1.47; two trials; 2,867 participants; moderate quality evidence). We attempted to categorise the participants according to the intensity of infection (none, light, moderate and heavy); however there were too few participants in each category to draw meaningful conclusions. The effect estimates from aggregate evidence were of similar size and direction as the IPD effect estimates. Table 9 reports the aggregate and IPD adjusted estimates for maternal hookworm intensity.

**Table 9:** Mass deworming on hookworm intensity (any infection)

Analysis	Effect estimates (RR and 95% CI)
Aggregate adjusted	0.39 (0.04, 3.93)
IPD adjusted	0.52 (0.18‐1.47)

### 11.4 Low birth weight

Two trials reported LBW suggesting no evidence of an impact of mass deworming on LBW (RR: 0.89, 95% CI: 0.67–1.18; two trials; 2,267 participants; moderate quality evidence). The effect estimates from aggregate evidence were of similar size and direction as the IPD effect estimates. Table 10 reports the aggregate and IPD adjusted estimates for LBW.

**Table 10:** Mass deworming on LBW

Analysis	Effect estimates (RR and 95% CI)
Aggregate adjusted	1.04 (0.79, 1.38)
IPD adjusted	0.89 (0.67, 1.18)

### 11.5 Preterm birth

Two trials reported preterm birth suggesting no evidence of an overall impact (RR: 0.69, 95% CI: 0.47–1.03; two trials; 2,707 participants; moderate quality evidence). The effect estimates from aggregate evidence were of similar size and direction as the IPD effect estimates. Table 11 reports the aggregate and IPD adjusted estimates for preterm birth.

**Table 11:** Mass deworming on preterm birth

Analysis	Effect estimates (RR and 95% CI)
Aggregate adjusted	0.84 (0.51, 1.39)
IPD adjusted	0.69 (0.47, 1.03)

## 12 EFFECT MODIFIER ANALYSES

Based on the availability of the data, we could only assess for effect modification by baseline *Trichiura* infection, maternal anaemia at baseline and maternal BMI at baseline. The overall model suggested a marginally significant impact of deworming on maternal anaemia (RR: 0.77, 95% CI: 0.73–0.81) with no evidence of impact on *Trichiura* infection, hookworm infection, LBW and preterm birth. The test for interaction was not statistically significant across the levels of *Trichiura* infection at baseline, maternal anaemia at baseline or maternal BMI at baseline for any of the outcomes. There was no evidence of effect modification by baseline *Trichiura* infection, maternal anaemia at baseline and maternal BMI at baseline. Table 12 depicts the estimates for full model and effect modification.

**Table 12:** Potential effect modification of mass deworming during pregnancy by baseline infection intensity, anaemia status and BMI

	Categories	Outcomes (RR with 95% CI)				
		Maternal anaemia	*Trichiura* infection	Hookworm infection	LBW	Preterm birth
**Mass deworming (overall)**		**0.77 (0.73–0.81)**	0.69 (0.42, 1.13)	0.52 (0.18, 1.47)	0.89 (0.67–1.18)	0.69 (0.47–1.03)
***Trichiura*** **Intensity at baseline**	Not infected	0.93 (0.80–1.09)	–	–	0.67 (0.43–1.04)	0.82 (0.50–1.36)
	Infected	0.81 (0.65‐1.02)	–	–	1.12 (0.68–1.86)	1.32 (0.68–2.55)
**Maternal Anaemia at baseline**	Normal	–	**0.65 (0.53–0.81)**	**0.51(0.42–0.62)**	0.80 (0.56–1.13)	**0.57 (0.36–0.92)**
	Anaemia (Hb < 11 g/dl)	–	**0.60 (0.46‐0.78)**	**0.56 (0.45–0.70)**	1.01 (0.68–1.49)	0.71 (0.41–1.22)
**Maternal BMI at baseline**	Normal	0.88 (0.77–1.01)	**0.61 (0.51–0.73)**	**0.49 (0.42–0.57)**	0.86 (0.65–1.15)	0.72 (0.48–1.09)
	Low ( < 18.5 kg/m2)	1.10 (0.74–1.63)	**1.53 (1.01–2.32)**	**0.36 (0.17–0.78)**	1.11 (0.47–2.64)	0.82 (0.20–3.34)

*Note*: Bold font indicates statitiscally significant estimates.

These findings are summarised in the summary of findings table (Table 13). All the outcomes were rated to be of moderate quality evidence. The evidence was downgraded by one level due to the study limitations since estimates are based on a sleceted sample received to conduct IPD.

**Table 13:** Summary of findings table

Mass deworming for STH and Schsitosomisis during pregnancy compared to placebo					
Population: Pregnant women					
Setting: Low‐ middle‐ income countries of Uganda, Tanzania and Philippnes					
Intervention: Mass deworming with any drug					
Comparison: Placebo					
		Aggregate evidence		IPD evidence	
Outcomes	No of Participants (Studies)	RR (95% CI)	Quality of the evidence (GRADE)	RR (95% CI)	Quality of the evidence (GRADE)
Maternal Anaemia	5216	0.94 (0.89–0.99)	⊕⊕⊕⊝	0.77 (0.73‐0.81)	⊕⊕⊕⊝
	(3 studies)		Moderate^a^		Moderate^a^
Maternal *T.Trichiura* intensity	2867	1.06 (0.87, 1.30)	⊕⊕⊕⊝	0.69 (0.42‐1.13)	⊕⊕⊕⊝
	(2 studies)		Moderate^a^		Moderate^a^
Maternal hookworm intensity	2867	0.39 (0.04, 3.93)	⊕⊕⊕⊝	0.52 (0.18‐1.47)	⊕⊕⊕⊝
	(2 studies)		Moderate^a^		Moderate^a^
LBW	2267	1.04 (0.79, 1.38)	⊕⊕⊕⊝	0.89 (0.67, 1.18)	⊕⊕⊕⊝
	(2 studies)		Moderate^a^		Moderate^a^
Preterm birth	2707	0.84 (0.51, 1.39)	⊕⊕⊕⊝	0.69 (0.47, 1.03)	⊕⊕⊕⊝
	(2 studies)		Moderate^a^		Moderate^a^

Abbreviations: CI, confidemce interval; LBW, low birthweight; RR, risk ratio; STH, soil transmitted helminths.

GRADE Working Group grades of evidence.

High quality: Further research is very unlikely to change our confidence in the estimate of effect.

Moderate quality: Further research is likely to have an important impact on our confidence in the estimate of effect and may change the estimate.

Low quality: Further research is very likely to have an important impact on our confidence in the estimate of effect and is likely to change the estimate.

Very low quality: We are very uncertain about the estimate.

^a^ Downgraded for study limitations ‐ obtained only a selected sample of IPD.

## 13 DISCUSSION

### 13.1 Summary of main results

This IPD meta‐analysis is based on the data from three trials with 5,957 participants. The effect estimates from aggregate evidence were of similar size and direction as the IPD effect estimates. Findings from this IPD suggest reduction in anaemia among pregnant women with mass deworming. There was no evidence of effect on any of the other outcomes including *Trichiura* infection, hookworm infection or any of the pregnancy outcomes including LBW and preterm birth. Findings of no impact of mass deworming on infection intensity could be attributable to the fact that majority of the study population in the included studies were either not infected or lightly infected which could have diluted the impact. Based on the availability of the data, we could only assess for effect modification by baseline *Trichiura* infection, maternal anaemia at baseline and maternal BMI at baseline. There was no evidence of effect modification by *Trichiura* intensity at baseline, maternal anaemia at baseline and maternal BMI at baseline; however we advise caution in interpreting these findings due to limited number of participants included in the analysis.

### 13.2 Overall completeness and applicability of evidence

Findings from this IPD analysis is based on 70% of the existing data deemed eligible for IPD (5957 participiants of 8515 participants). The studies included in this review were conducted among pregnant women in LMIC settings. One of the three trials included in the IPD analysis provided daily iron folate supplements (36 mg iron; 5 mg folate) along with the deworming drugs.

We conducted an extensive search of electronic databases, with advice from the Campbell Collaboration International Development Group information scientist. We screened 23406 articles and updated this search to March 2018. We report the systematic review according to the reporting guidelines for IPD meta‐analysis (PRISMA‐IPD). We published and followed an *a priori* protocol (Salam et al). Our systematic review and IPD analysis was approved by the Research Ethics Boards at SickKids. We developed a data sharing agreement that was signed by all studies that contributed data. Study authors were invited to join the Investigator's Collaborative, participate in meetings and contribute to the final report. Our process and conduct of the IPD was driven by consultation with our expert Advisory board which included statistical, parasitology and nutrition expertise.

### 13.3 Quality of the evidence

The trials included in the IPD were judged to be of fairly good quality. All of the included studies were judged to be at low risk of bias for blinding of participants, personnel and outcome assessor; and selective reportong. One of the included studies was judged to be at high risk of bias for allocation concealment while two studies were at high risk for attrition bias. The overall outcome quality was judged to ‘moderate’ based on the GRADE criteria. The outcome quality was downgraded due to study limitations since the estimates are based on selected sample eligible for IPD.

### 13.4 Limitations and potential biases in the review process

Despite of receiving majority of the existing data (70%) to conduct IPD, there were a few limitations. One limitation of this review is that we did not receive data from all eligible studies. Another limitation is that we were unable to assess effect modification by pre‐idnetified effect modifiers. The trials did not capture many of the variables of interest that restricted our analysis. Very few trials reported outcomes according to the baseline level of infection intensities and hence those conclusions could not be drawn. In terms of the infection intensities, the population studied were either not infected or lightly infected and hence it was difficult to categorise the sample according to the intensity of infection and have meaningful estimates. Trials did not report baseline data on the individual and environmental level effect modifiers and hence it was difficult to assess the effect modification. Variables like socio‐economic status were least studied and where reported, had different definitions and hence could not be accounted for. None of the included studies assessed any co‐interventions including WASH practices and hence the impact of co‐interventions could not be assessed. We could not assess for publication bias given the small number of included studies; however, considering the small universe of studies in the domain, the issues related to publication and small study sizes cannot be ignored.

### 13.5 Agreements and disagreements with other studies or reviews

The most recent Cochrane meta‐analysis (Salam et al., 2015) on deworming for STH during pregnancy concluded that there was insufﬁcient evidence to recommend deworming for STH. This review also highlighted the need for future well‐designed, large scale RCTs to establish the beneﬁt. These findings were based on four trials including 4265 participants. This review has some differences compared to our review. The inclusion criteria for this Cochrane review was limited to deworming for STH alone while our IPD meta‐analysis also included trials with deworming for schistosomiasis. The Cochrane review reported no impact of mass deworming for STH on maternal anaemia while findings from our review suggests reduction in maternal anaemia associated with mass deworming,

### 13.6 Implications for policy

This systematic review and IPD suggest that mass deworming reduces maternal anaemia with moderate quality evidence. The policy implications are that, even in high‐prevalence areas, deworming alone is insufficient to achieve improvements in all maternal and newborn health outcomes. These findings reinforce that it is essential to focus on sustainable development to address the other factors such as poor sanitation, food insecurity and malnutrition. Mass deworming should be bundled as part of these packages to improve range of maternal and newborn health outcomes.

### 13.7 Implications for research

There is a need to evaluate mass deworming for STH and schistosomiasis during pregnancy in large scale programmatic settings. Future impact evaluations should attempt to measure various individual and environmental factors that could potentially effect the impact of mass deworming. Future program evaluations should also assess the long term impact of mass deworming on birth and infant health outcomes along with the maternal health outcomes. There is an urgent need for open data from all research studies. The quality of evidence is rated as moderate for our findings. Further research on maternal baseline worm intensities and birth outcomes could change our findings.

## PLANS FOR UPDATING THE REVIEW

This review will be updated if funds become available.

## SOURCES OF SUPPORT

This review is funded by the Bill and Melinda Gates Foundation (Funding reference number: OPP1140742).

## DECLARATIONS OF INTEREST

Michelle Gaffey, Robert Black, Deidre Hollingsworth, Sue Horton, Rehana Salam, and Simon Cousens have no conflict of interest, financial or otherwise that may influence judgments made in this review.

Celia Holland is a co‐author and principal investigator on a randomised trial of deworming in children: Kirwan et al 2009 (Kirwan, P., Asaolu, S. O., Molloy, S. F., Abiona, T. C., Jackson, A. L. & Holland, C. V. (2009). Patterns of soil‐transmitted helminth infection and impact of four‐monthly albendazole treatments in preschool children from semi‐urban communities in Nigeria: a double‐blind placebo‐controlled randomised trial. BMC infectious diseases, 9(1), 20.)

Vivian Welch and Zulfi Bhutta are authors of the Campbell systematic review and network meta‐analysis of mass deworming for children (Welch, Ghogomu et al. 2016).

Vivian Welch is editor‐in‐chief of the Campbell Collaboration.

## AUTHORS CONTRIBUTION

Rehana A Salam and Michelle Gaffey collated the data for IPD; Simon Cousens supervised the statistical analysis; Paul Arora, Vivian Welch, Robert Black, Celia Holland, Deirdre Hollingsworth, Sue Horton Sanjay Wijesekera, Philippa Middleton, Maria Makrides and Zulfiqar A Bhutta provided overall feedback at each stage.

## DIFFERENCE BETWEEN PROTOCOL AND REVIEW

We could not conduct the following analyses as planned due to limited number of included studies

1. We could not conduct planned pair‐wise comaprisons for one deworming drug versus other deworming drug or a combination of deworming drugs.
2. We could not assess for publication bias as planned due to < 10 studies included.
3. We could not conduct the planned subgroup analysis and effect modification.

## APPENDIX A ADDITIONAL TABLE

See Additional Table 1.

**Table 1:** PRISMA‐IPD reporting checklists

PRISMA‐IPD Checklist of items to include when reporting a systematic review and meta‐analysis of individual participant data (IPD) PRISMA‐IPD			
Section/topic Title	Item No	Checklist item	Reported on page
Title	1	Identify the report as a systematic review and meta‐analysis of individual participant data.	1
**Abstract**			
Structured summary	2	Provide a structured summary including as applicable:	2–4
		**Background**: state research question and main objectives, with information on participants, interventions, comparators and outcomes.	
		**Methods**: report eligibility criteria; data sources including dates of last bibliographic search or elicitation, noting that IPD were sought; methods of assessing risk of bias.	
		**Results**: provide number and type of studies and participants identified and number (%) obtained; summary effect estimates for main outcomes (benefits and harms) with confidence intervals and measures of statistical heterogeneity. Describe the direction and size of summary effects in terms meaningful to those who would put findings into practice.	
		**Discussion:** state main strengths and limitations of the evidence, general interpretation of the results and any important implications.	
		**Other:** report primary funding source, registration number and registry name for the systematic review and IPD meta‐analysis.	
**Introduction**			
Rationale	3	Describe the rationale for the review in the context of what is already known.	5–9
Objectives	4	Provide an explicit statement of the questions being addressed with reference, as applicable, to participants, interventions, comparisons, outcomes and study design (PICOS). Include any hypotheses that relate to particular types of participant‐level subgroups.	9
**Methods**			
Protocol and registration	5	Indicate if a protocol exists and where it can be accessed. If available, provide registration information including registration number and registry name. Provide publication details, if applicable.	9
Eligibility criteria	6	Specify inclusion and exclusion criteria including those relating to participants, interventions, comparisons, outcomes, study design and characteristics (e.g. years when conducted, required minimum follow‐up). Note whether these were applied at the study or individual level i.e. whether eligible participants were included (and ineligible participants excluded) from a study that included a wider population than specified by the review inclusion criteria. The rationale for criteria should be stated.	10
Identifying studies ‐ information sources	7	Describe all methods of identifying published and unpublished studies including, as applicable: which bibliographic databases were searched with dates of coverage; details of any hand searching including of conference proceedings; use of study registers and agency or company databases; contact with the original research team and experts in the field; open adverts and surveys. Give the date of last search or elicitation.	11
Identifying studies ‐ search	8	Present the full electronic search strategy for at least one database, including any limits used, such that it could be repeated.	40–44
Study selection processes	9	State the process for determining which studies were eligible for inclusion.	10–11
Data collection processes	10	Describe how IPD were requested, collected and managed, including any processes for querying and confirming data with investigators. If IPD were not sought from any eligible study, the reason for this should be stated (for each such study).	12–13
		If applicable, describe how any studies for which IPD were not available were dealt with. This should include whether, how and what aggregate data were sought or extracted from study reports and publications (such as extracting data independently in duplicate) and any processes for obtaining and confirming these data with investigators.	
Data items	11	Describe how the information and variables to be collected were chosen. List and define all study level and participant level data that were sought, including baseline and follow‐up information. If applicable, describe methods of standardising or translating variables within the IPD datasets to ensure common scales or measurements across studies.	12–15
IPD integrity	A1	Describe what aspects of IPD were subject to data checking (such as sequence generation, data consistency and completeness, baseline imbalance) and how this was done.	13
Risk of bias assessment in individual studies.	12	Describe methods used to assess risk of bias in the individual studies and whether this was applied separately for each outcome. If applicable, describe how findings of IPD checking were used to inform the assessment. Report if and how risk of bias assessment was used in any data synthesis.	12
Specification of outcomes and effect measures	13	State all treatment comparisons of interests. State all outcomes addressed and define them in detail. State whether they were pre‐specified for the review and, if applicable, whether they were primary/main or secondary/additional outcomes. Give the principal measures of effect (such as risk ratio, hazard ratio, difference in means) used for each outcome.	10–11
Synthesis methods	14	Describe the meta‐analysis methods used to synthesise IPD. Specify any statistical methods and models used. Issues should include (but are not restricted to):	12–15
		Use of a one‐stage or two‐stage approach.	
		How effect estimates were generated separately within each study and combined across studies (where applicable).	
		Specification of one‐stage models (where applicable) including how clustering of patients within studies was accounted for.	
		Use of fixed or random effects models and any other model assumptions, such as proportional hazards.	
		How (summary) survival curves were generated (where applicable).	
		Methods for quantifying statistical heterogeneity (such as I^2^ and τ^2^).	
		How studies providing IPD and not providing IPD were analysed together (where applicable).	
		How missing data within the IPD were dealt with (where applicable).	
Exploration of variation in effects	A2	If applicable, describe any methods used to explore variation in effects by study or participant level characteristics (such as estimation of interactions between effect and covariates). State all participant‐level characteristics that were analysed as potential effect modifiers, and whether these were pre‐specified.	14–15
Risk of bias across studies	15	Specify any assessment of risk of bias relating to the accumulated body of evidence, including any pertaining to not obtaining IPD for particular studies, outcomes or other variables.	12
Additional analyses	16	Describe methods of any additional analyses, including sensitivity analyses. State which of these were pre‐specified.	15
**Results**			
Study selection and IPD obtained	17	Give numbers of studies screened, assessed for eligibility, and included in the systematic review with reasons for exclusions at each stage. Indicate the number of studies and participants for which IPD were sought and for which IPD were obtained. For those studies where IPD were not available, give the numbers of studies and participants for which aggregate data were available. Report reasons for non‐availability of IPD. Include a flow diagram.	16–1722
Study characteristics	18	For each study, present information on key study and participant characteristics (such as description of interventions, numbers of participants, demographic data, unavailability of outcomes, funding source, and if applicable duration of follow‐up). Provide (main) citations for each study. Where applicable, also report similar study characteristics for any studies not providing IPD.	19–20
IPD integrity	A3	Report any important issues identified in checking IPD or state that there were none.	22–24
Risk of bias within studies	19	Present data on risk of bias assessments. If applicable, describe whether data checking led to the up‐weighting or down‐weighting of these assessments. Consider how any potential bias impacts on the robustness of meta‐analysis conclusions.	21
Results of individual studies	20	For each comparison and for each main outcome (benefit or harm), for each individual study report the number of eligible participants for which data were obtained and show simple summary data for each intervention group (including, where applicable, the number of events), effect estimates and confidence intervals. These may be tabulated or included on a forest plot.	
Results of syntheses	21	Present summary effects for each meta‐analysis undertaken, including confidence intervals and measures of statistical heterogeneity. State whether the analysis was pre‐specified, and report the numbers of studies and participants and, where applicable, the number of events on which it is based.	25–27
		When exploring variation in effects due to patient or study characteristics, present summary interaction estimates for each characteristic examined, including confidence intervals and measures of statistical heterogeneity. State whether the analysis was pre‐specified. State whether any interaction is consistent across trials.	
		Provide a description of the direction and size of effect in terms meaningful to those who would put findings into practice.	
Risk of bias across studies	22	Present results of any assessment of risk of bias relating to the accumulated body of evidence, including any pertaining to the availability and representativeness of available studies, outcomes or other variables.	21
Additional analyses	23	Give results of any additional analyses (e.g. sensitivity analyses). If applicable, this should also include any analyses that incorporate aggregate data for studies that do not have IPD. If applicable, summarise the main meta‐analysis results following the inclusion or exclusion of studies for which IPD were not available.	27
**Discussion**			
Summary of evidence	24	Summarise the main findings, including the strength of evidence for each main outcome.	28–30
Strengths and limitations	25	Discuss any important strengths and limitations of the evidence including the benefits of access to IPD and any limitations arising from IPD that were not available.	28–30
Conclusions	26	Provide a general interpretation of the findings in the context of other evidence.	28–30
Implications	A4	Consider relevance to key groups (such as policy makers, service providers and service users). Consider implications for future research.	28–30
**Funding**			
Funding	27	Describe sources of funding and other support (such as supply of IPD), and the role in the systematic review of those providing such support.	35

**A1 – A3 denote new items that are additional to standard PRISMA items. A4 has been created as a result of re‐arranging content of the standard PRISMA statement to suit the way that systematic review IPD meta‐analyses are reported.**

© Reproduced with permission of the PRISMA IPD Group, which encourages sharing and reuse for non‐commercial purposes

## APPENDIX B SEARCH STRATEGY

Database: Ovid MEDLINE(R) In‐Process & Other Non‐Indexed Citations and Ovid MEDLINE(R).

1. flukes.tw.
2. platyhelminth*.tw.
3. whipworm*.tw.
4. whip worm*.tw.
5. hookworm*.tw.
6. hookworm*.tw.
7. hook worm*.tw.
8. roundworm*.tw.
9. round worm*.tw.
10. geohelminth*.tw.
11. ancylostoma*.tw.
12. Necator*.tw.
13. Ascaris.tw.
14. Ascaridida.tw.
15. Ancylostoma.tw.
16. Necator americanus.tw.
17. Trichuris.tw.
18. Trichuroidea.tw.
19. Adenophorea.tw.
20. Enoplida.tw.
21. Ascaridida.tw.
22. Platyhelminth*.tw.
23. Rotifera.tw.
24. trichuriasis.tw.
25. ascariasis.tw.
26. ancylostomiasis.tw.
27. ascarid*.tw.
28. schistosom*.tw.
29. bilharziosis.tw.
30. bilharzia*.tw.
31. exp Schistosoma/
32. or/1‐31
33. Albendazole/
34. Mebendazole/
35. exp Piperazines/
36. Levamisole/
37. exp Pyrantel/
38. Ivermectin/
39. exp Anthelmintics/
40. Ivermectin.tw.
41. Albendazole.tw.
42. Mebendazole.tw.
43. Piperazine*.tw.
44. Levamisole.tw.
45. pyrantel.tw.
46. tiabendazole.tw.
47. anthelmint*.tw.
48. Anticestodal.tw.
49. Antiplatyhelmintic.tw.
50. Anti‐platyhelmintic.tw.
51. Albendazole.tw.
52. Dichlorophen.tw.
53. Niclosamide.tw.
54. Bithionol.tw.
55. Diamfenetide.tw.
56. Nitroxinil.tw.
57. Oxyclozanide.tw.
58. Rafoxanide.tw.
59. Schistosomicid*.tw.
60. Antimony Potassium Tartrate.tw.
61. Antimony Sodium Gluconate.tw. = 62 Hycanthone.tw.
62. Lucanthone.tw.
63. Niridazole.tw.
64. Oxamniquine.tw.
65. Praziquantel/
66. Trichlorfon/
67. metrifonate.tw.
68. Artemisinins/
69. (artesunate or artemether).tw.
70. or/34‐72
71. (deworm* or de‐worm*).tw.
72. exp Anthelmintics/ or (anthelmint* or antihelmint*).tw.
73. 72 or 73
74. Pregnant Women/ or Pregnancy/ or Pregnancy Complications, Parasitic/ pregnant wom n .tw.
75. 32 and 71
76. 74 or 76
77. 75 and 77

Database: Embase Classic + Embase

1. whipworm*.tw.
2. whip worm*.tw.
3. hookworm*.tw.
4. hookworm*.tw.
5. hook worm*.tw.
6. roundworm*.tw.
7. round worm*.tw.
8. pinworm*.tw.
9. pin worm*.tw.
10. flukes.tw.
11. geohelminth*.tw.
12. ancylostoma.tw.
13. Necator*.tw.
14. Ascaris.tw.
15. Ascaridida.tw.
16. Ancylostoma.tw.
17. Necator americanus.tw.
18. Enterobius.tw.
19. Oxyuroidea.tw.
20. Oxyurida.tw.
21. Trichuris.tw.
22. Trichuroidea.tw.
23. Capillaria.tw.
24. Trichinella.tw.
25. Strongyloid*.tw.
26. Oesophagostomum.tw.
27. Oesophagostomiasis.tw.
28. Acanthocephala.tw.
29. Adenophorea.tw.
30. Enoplida.tw.
31. Secernentea.tw.
32. Ascaridida.tw.
33. Rhabditida.tw.
34. Cestoda.tw.
35. Trematod*.tw.
36. Turbellaria.tw.
37. Platyhelminth*.tw.
38. Rotifera.tw.
39. trichuriasis.tw.
40. ascariasis.tw.
41. trichinellosis.tw.
42. Trichostrongyloidiasis.tw.
43. ancylostomiasis.tw.
44. enterobiasis.tw.
45. cestode*.tw.
46. trematode*.tw.
47. ascarid*.tw.
48. schistosomiasis.tw.
49. Schistosoma*.tw.
50. or/1‐49
51. Albendazole/
52. Mebendazole/
53. exp Piperazines/
54. Levamisole/
55. exp Pyrantel/
56. Ivermectin/
57. exp Anthelmintics/
58. Ivermectin.tw.
59. Albendazole.tw.
60. Mebendazole.tw.
61. Piperazine*.tw.
62. Levamisole.tw.
63. pyrantel.tw.
64. tiabendazole.tw.
65. anthelmint*.tw.
66. *Antiplatyhelmintic Agents/
67. Anticestodal.tw.
68. Antiplatyhelmintic.tw.
69. Anti‐platyhelmintic.tw.
70. Albendazole.tw.
71. Dichlorophen.tw.
72. Niclosamide.tw.
73. Bithionol.tw.
74. Diamfenetide.tw.
75. Nitroxinil.tw.
76. Oxyclozanide.tw.
77. Rafoxanide.tw.
78. Schistosomicide*.tw.
79. Antimony Potassium Tartrate.tw.
80. Antimony Sodium Gluconate.tw.
81. Hycanthone.tw.
82. Lucanthone.tw.
83. Niridazole.tw.
84. Oxamniquine.tw.
85. or/51‐84
86. (deworm* or de‐worm*).tw.
87. anthelmint*.tw.
88. anthelmintic/
89. or/86‐88
90. pregnant wom*n .tw.
91. (woman or women).tw.
92. pregnan*.tw.
93. or/90‐92
94. 50 and 85
95. 94 or 89
96. 95 and 93

## APPENDIX C EXTRACTION SHEET

Information was extracted for the following characteristics from all the included studies:

1. Study no
2. Study ID
3. Study design
4. Country
5. Settings
6. Sample size
7. Intervention group details (Dosing, frequency, delivered by)
8. Control group details
9. Follow‐up
10. Baseline characteristics reported
11. Outcomes reported
